# Supplementary material for: Tumor cell-based liquid biopsy using high-throughput microfluidic enrichment of entire leukapheresis product
Source: Nat Commun. 2025 Jan 2;16:32. doi: 10.1038/s41467-024-55140-x (PMC11696112; doi:10.1038/s41467-024-55140-x)
Supplement: Supplementary file 1 — Supplementary Information File [file 41467_2024_55140_MOESM1_ESM.pdf]

Supplementary Information File

Tumor cell-based liquid biopsy using high-throughput microfluidic enrichment of entire leukapheresis product

Avanish Mishra[1,2 #], Shih-Bo Huang[2,3 #], Taronish Dubash[2], Risa Burr[2], Jon F. Edd[1,2], Ben S. Wittner[2], Quinn E. Cunneely[1,2], Victor R. Putaturo[1,2], Akansha Deshpande[1,2], Ezgi Antmen[1,2], Kaustav A. Gopinathan[1,2], Keisuke Otani[2,4], Yoshiyuki Miyazawa[2,4], Ji Eun Kwak[2], Sara Y. Guay[2], Justin Kelly[2,4], John Walsh[1,2], Linda T. Nieman[2], Isabella Galler[5], PuiYee Chan[5], Michael S. Lawrence[2,6,7], Ryan J. Sullivan[5], Aditya Bardia[8], Douglas S. Micalizzi[2,5], Lecia V. Sequist[5], Richard J. Lee[5], Joseph W. Franses[5], David T. Ting[2,5], Patricia A. R. Brunker[6], Shyamala Maheswaran[2], David T. Miyamoto[2,4,7*], Daniel A. Haber[2,3,5*], and Mehmet Toner[1,9*]

[1]Center for Engineering in Medicine and Surgery, Massachusetts General Hospital and Harvard Medical School, Boston, Massachusetts, 02114, USA

[2]Krantz Family Center for Cancer Research, Massachusetts General Hospital Cancer Center and Harvard Medical School, Charlestown, Massachusetts, 02129, USA

[3]Howard Hughes Medical Institute, Bethesda, Maryland, 20815, USA

[4]Department of Radiation Oncology, Massachusetts General Hospital and Harvard Medical School, Boston, Massachusetts, 02114, USA

[5]Division of Hematology Oncology, Massachusetts General Hospital Cancer Center and Harvard Medical School, Boston, Massachusetts, 02114, USA

[6]Department of Pathology, Massachusetts General Hospital and Harvard Medical School, Boston, Massachusetts, 02114, USA

[7]Broad Institute of MIT and Harvard, Cambridge, MA 02142, USA

[8]Hematology/Oncology, University of California, Los Angeles (current affiliation)

[9]Shriners Children’s Boston, Massachusetts, 02114, USA.

# Equal contribution

* Corresponding authors

Mehmet Toner (mehmet_toner@hms.harvard.edu)

Daniel A. Haber (DHABER@mgh.harvard.edu)

David T. Miyamoto (dmiyamoto@mgh.harvard.edu)

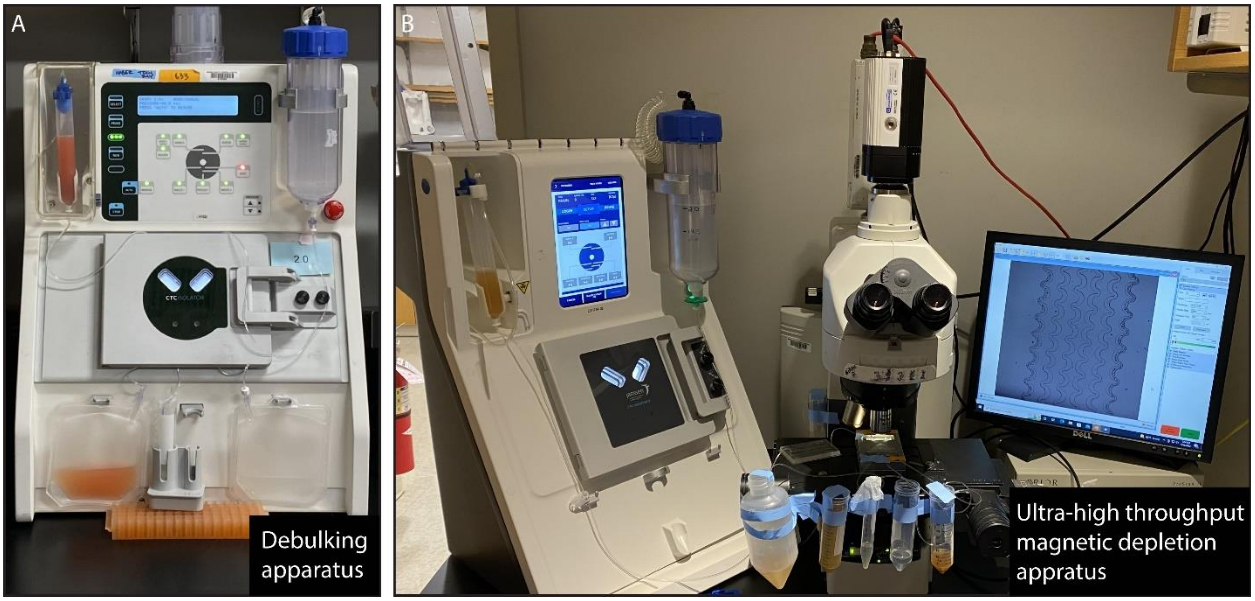

Supplementary Figure 1: The apparatus used for microfluidic isolation of CTCs from leukopaks. (A) The debulking apparatus consists of a pressure source to modulate sample and buffer flow rates. It allows for the collection of WBCs and CTCs from leukopaks into a clean buffer while removing RBCs, platelets, and plasma. (B) The magnetic depletion apparatus uses an aluminum adapter to hold the magnets and the magnetic sorter securely while utilizing the same pressure source as the debulking system. The ultrahigh-throughput magnetic sorter removes magnetically tagged WBCs away from the unmanipulated CTCs.

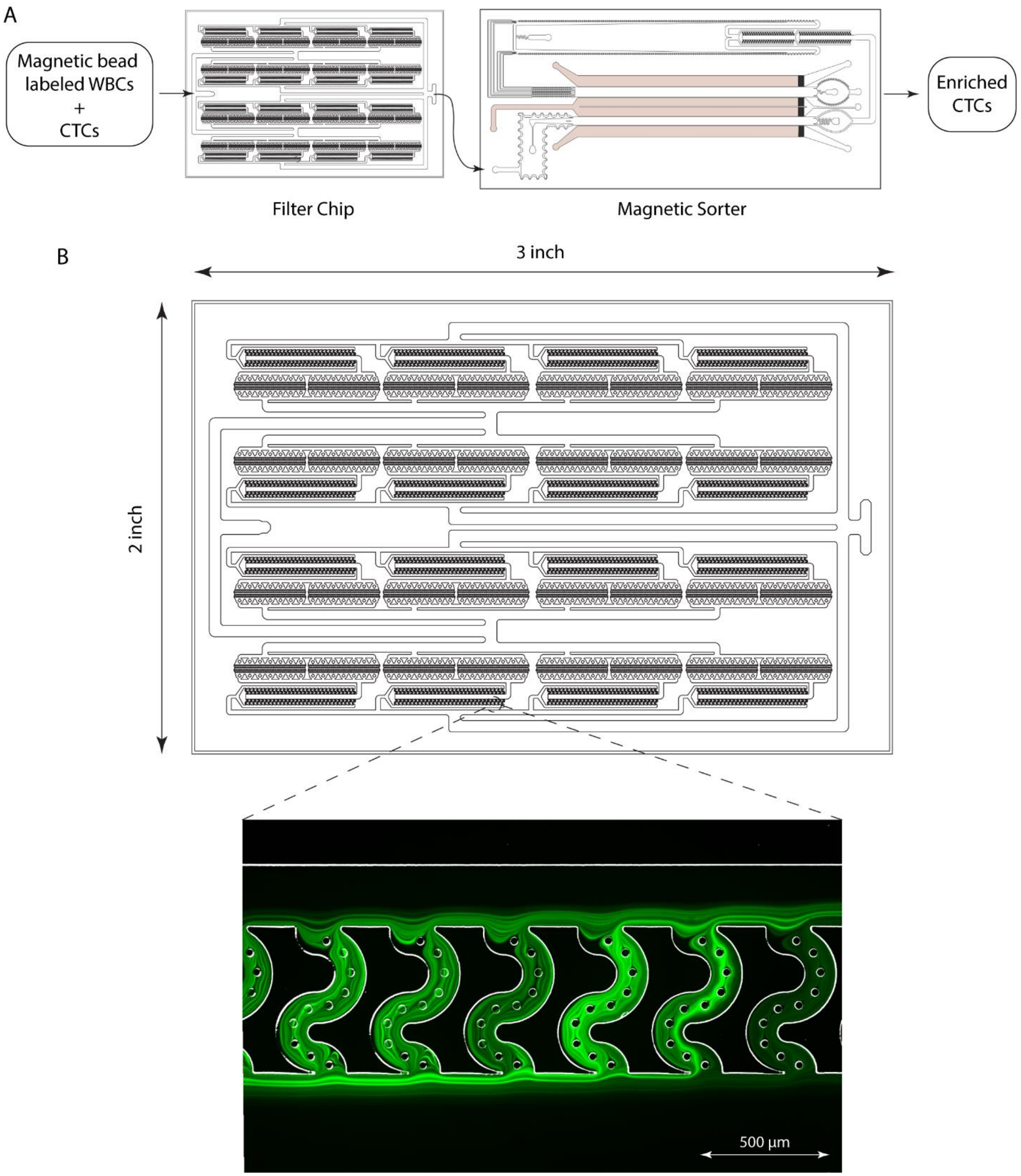

Supplementary Figure 2: Filter chip. (A) This chip was used in-line with the magnetic sorter for removing large clots or aggregates of cells. (B) A schematic diagram of the filter chip. The inset shows streak images of fluorescently labeled WBCs flowing through the filter (n = 6).

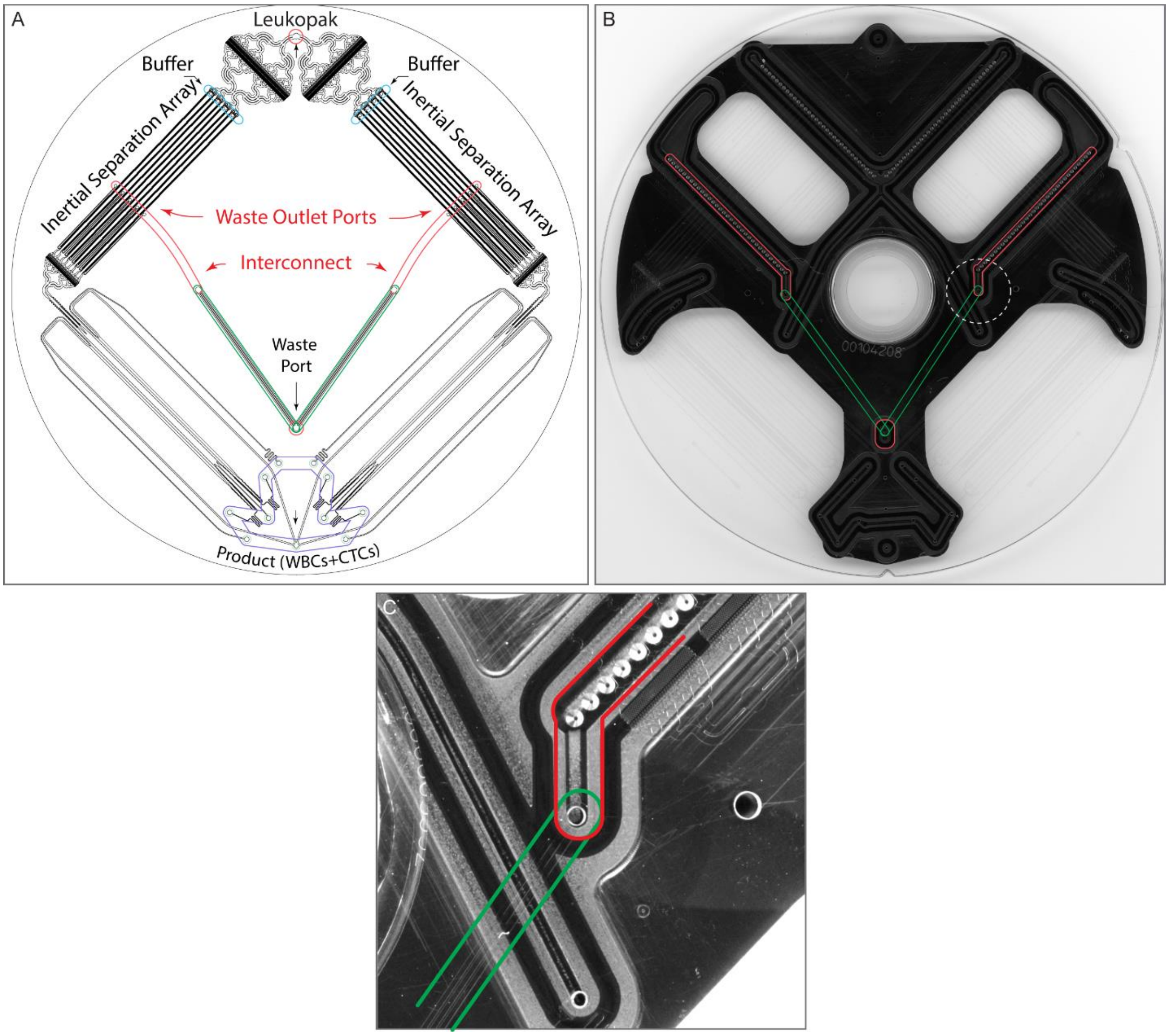

Supplementary Figure 3: Interconnect channels allow outputs from multiple channels to be combined into a single outlet port. In the debulking chip, a third (black plastic) layer consists of interconnect channels, collecting waste outlets of 16 inertial separation arrays through 10 holes. This black plastic layer was fused to the top layer of the injection molded microfluidic plastic chip, creating a single monolithic chip. (A) Schematic diagram of the debulking chip with a layout of interconnecting channels. (B) An image of the debulking chip with a third layer made of black injection molded plastic. (C) A magnified image of an interconnecting channel (shown in green) collecting output from the multiple holes (shown in red).

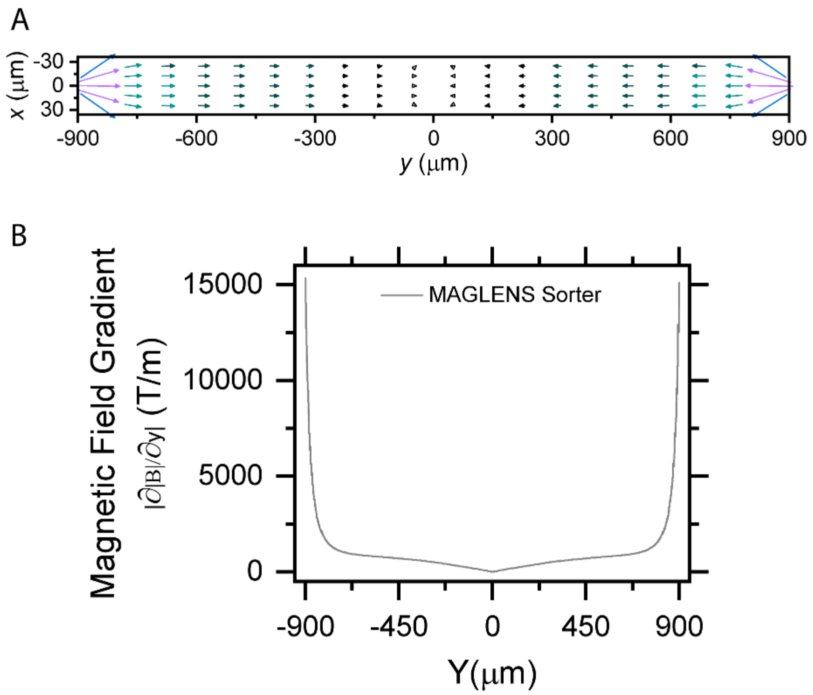

Supplementary Figure 4: (A) Vector plot of magnetic gradient in the sorting channel. Magnetic forces vanish in the center of the channel, creating an inherently safe design for sorting billions of cells without clogging. (B) A high magnetic gradient created by magnetic lenses.

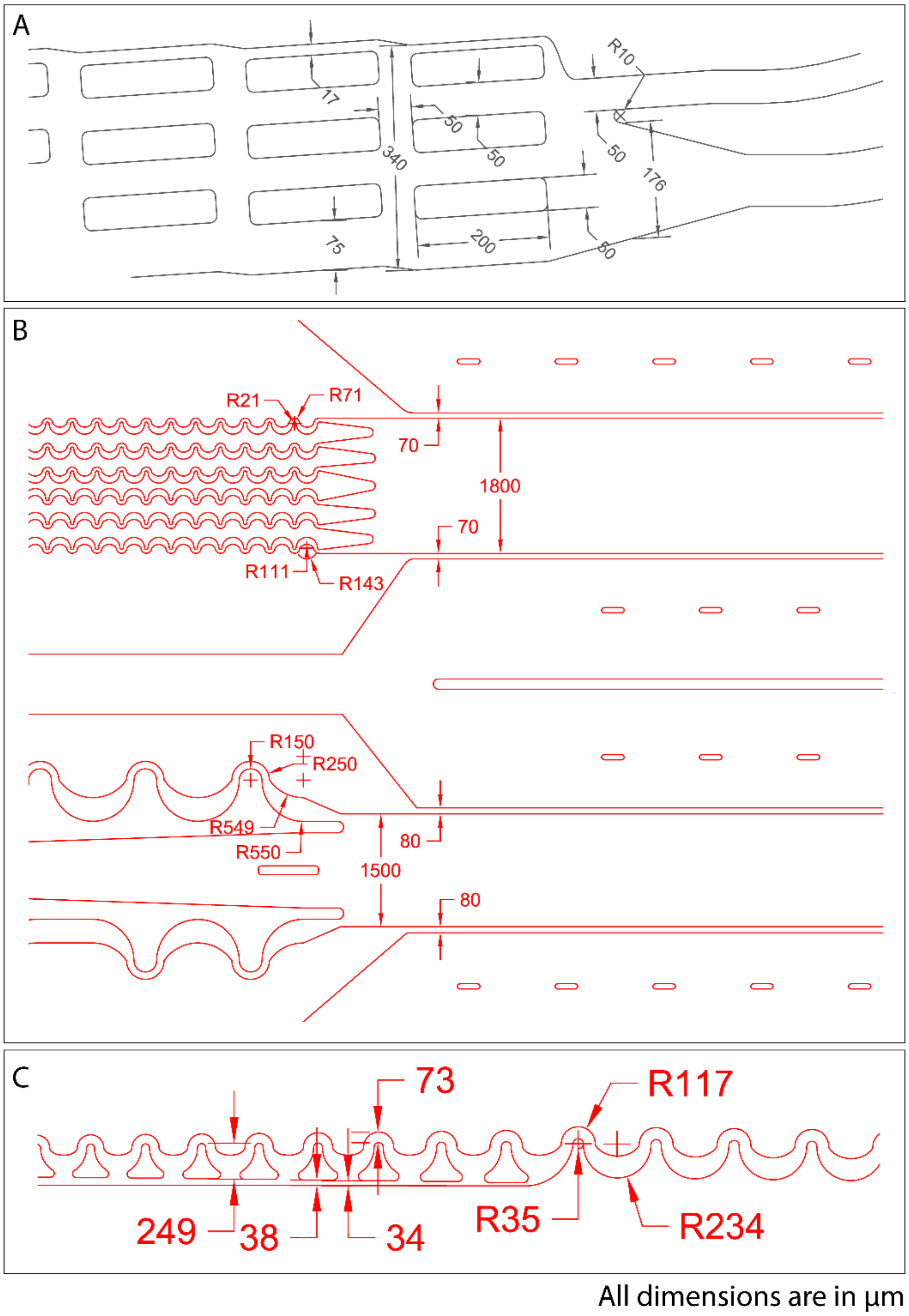

Supplementary Figure 5: Critical dimensions of inertial separation array in debulking chip (A), sorting channels (B), and inertial cell concentrator in MAGLENS chip (C). All dimensions are in µm.

A

B

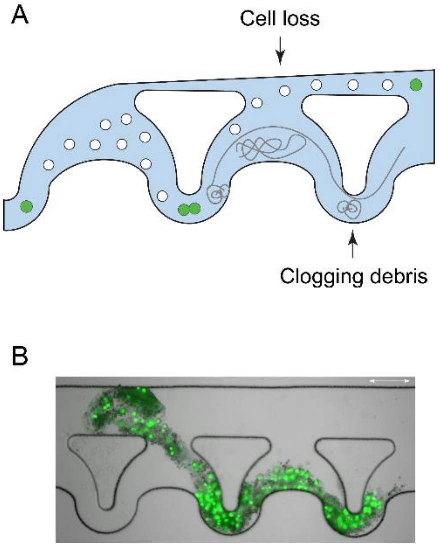

Supplementary Figure 6: Clogging of an inertial concentrator by neutrophil extracellular traps (NETS) in the absence of DNAse. (A) NETS wrap around siphoning pillars and clog the channels, resulting in cell loss. (B) An image of a clogged microfluidic concentrator (n = 5). Cells are labeled with a fluorescent Dye Cycle Green marker. The scale bar is 100 µm.

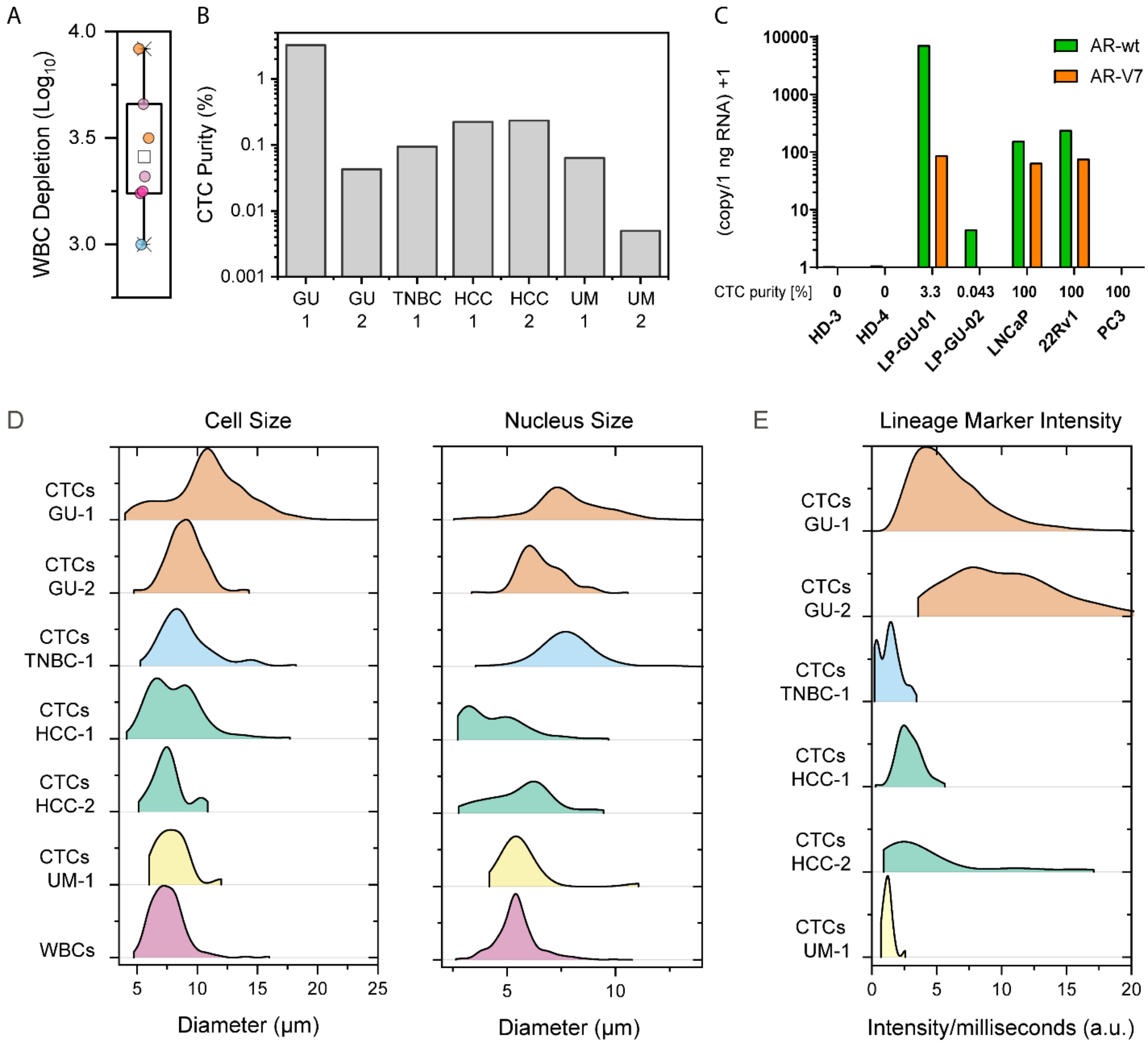

Supplementary Figure 7: (A) WBC depletion after LPCTC-iChip processing of leukopak samples from various cancers. This approach, on average, results in the removal of 99.96% of WBCs while recovering 100 to 58125 CTCs. (B) Bar graph showing CTC purity following enrichment. (C) Droplet digital RNA-PCR (ddPCR) analysis was performed on CTC products from two prostate cancer cases, designated GU-1 and GU-2. This analysis quantified the expression of wild-type androgen receptor (AR-wt) and AR variant 7 (AR-V7) in the context of normal blood cells. Notably, GU-1 CTCs expressed both AR-wt and AR-V7, while GU-2 CTCs expressed only AR-wt. Leukocytes from the blood of two healthy donors and the AR-null prostate cancer cell line PC-3 served as negative controls. Positive controls were provided by cultured AR-positive prostate cancer cell lines (LNCaP and 22Rv1). Tumor fractions are indicated beneath the corresponding bars in the analysis. (D) Measured whole cell and nucleus diameters of individual CTCs, compared with WBCs. (E) Variation across individual CTCs from cases GU-1, GU-2, TNBC-1, HCC-1, HCC-2, and UM-1 in their intensity of staining for the combined lineage markers.

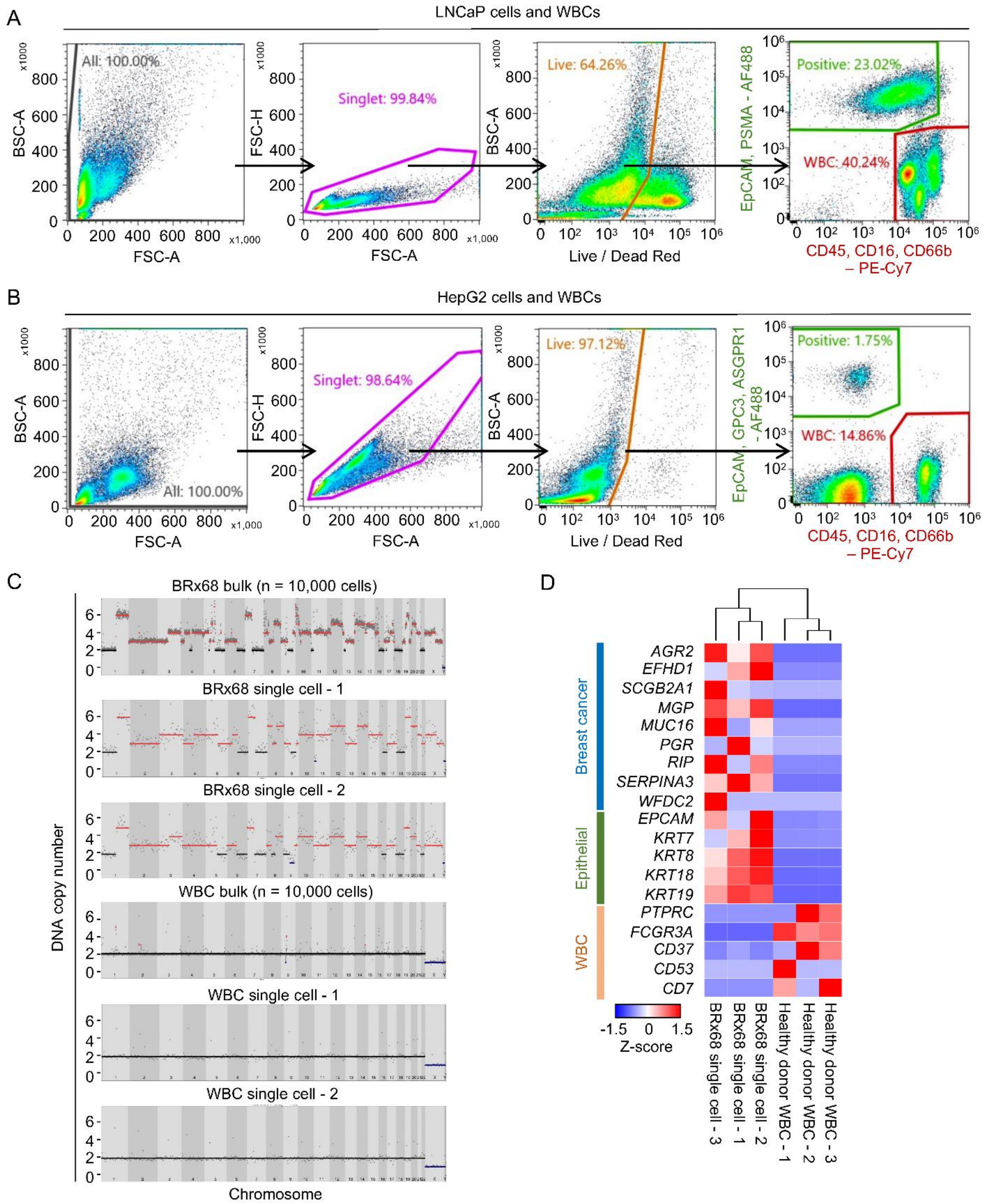

Supplementary Figure 8: Development of flow cytometry antibody panels to isolate single CTCs from prostate and hepatocellular cancer (HCC) leukopaks and associated single-cell analysis. (A) Representative FACS sorting using a customized antibody panel including Alexa Fluorophore (AF) 488-conjugated antibodies against EpCAM and PSMA and PE-Cy7-conjugated antibodies against WBC markers CD45, CD16, and CD66b. A mixture of cells from the prostate cancer cell line LNCaP and healthy donor WBCs was used to validate

this panel. (B) Representative FACS sorting using customized antibody panels, including AF488-conjugated antibodies against EpCAM, GPC3, and ASGPR1 and PE-Cy7-conjugated antibodies against WBC markers CD45, CD16, and CD66b. A mixture of cells from the liver cancer cell line HepG2 and healthy donor WBCs was used to validate this panel. (C) Representative DNA copy-number variation (CNV) analysis in bulk and in single cells derived from the breast CTC culture BRx68, compared with diploid genomes in bulk and single leukocytes. Ginkgo was used for DNA copy number analysis from whole genome sequencing data. (D) Unsupervised hierarchical clustering of z-transformed heatmap of gene expression in single-cell RNA-seq data. The cultured BRx68 CTCs show high expression of epithelial and breast cancer lineage markers and the absence of leukocyte (WBC) markers. Single WBCs that persisted after processing through the microfluidic device are shown as negative controls. Pearson’s correlation matrix was used for hierarchical clustering.

A

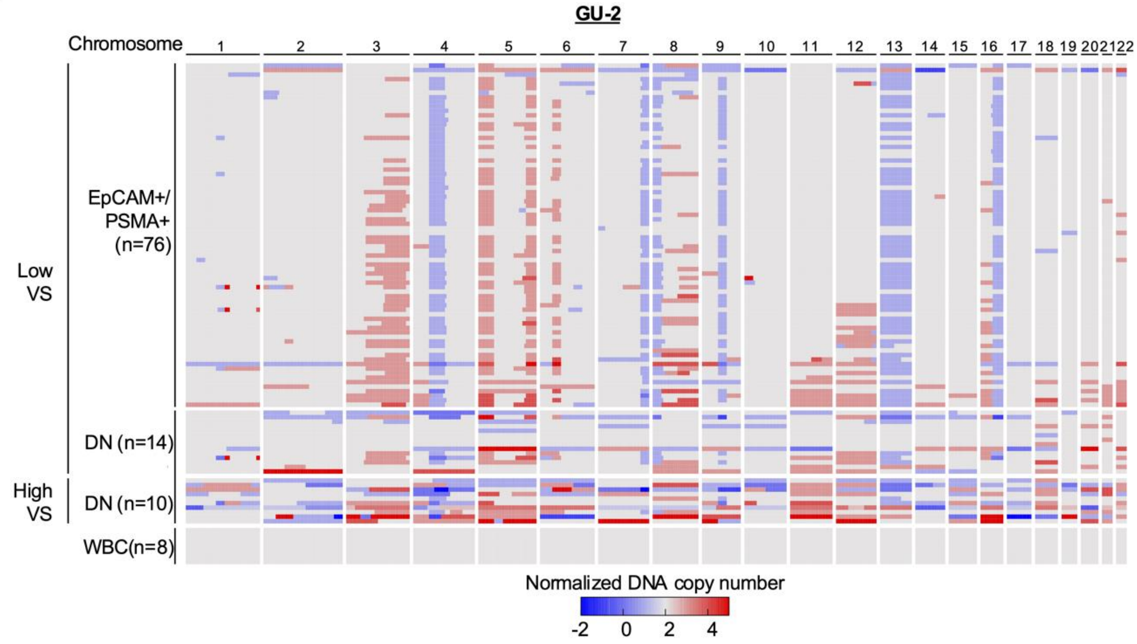
GU-2
Chromosome
1
2
3
4
5
6
7
8
9
10
11
12
13
14
15
16
17
18
19
20
21
22
Low VS
EpCAM+/ PSMA+ (n=76)
DN (n=14)
High VS
DN (n=10)
WBC(n=8)
Normalized DNA copy number
-2
0
2
4

B

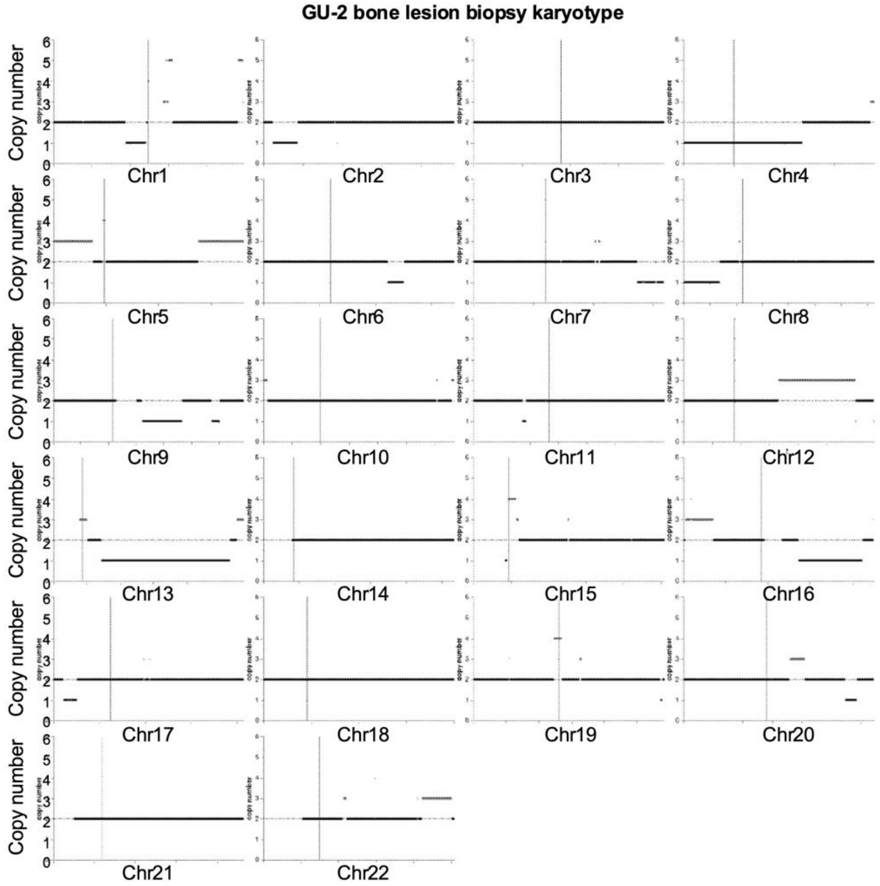
GU-2 bone lesion biopsy karyotype
Copy number
Chr1
Chr2
Chr3
Chr4
Chr5
Chr6
Chr7
Chr8
Chr9
Chr10
Chr11
Chr12
Chr13
Chr14
Chr15
Chr16
Chr17
Chr18
Chr19
Chr20
Chr21
Chr22

Supplementary Figure 9: Single-cell whole genome sequencing and DNA copy number analyses. (A) Normalized heatmap showing clustering of single-cell CNVs across the genome in CTCs (n=100) from an mCRPC patient, GU-2. All CTCs exhibit shared core chromosomal alterations, and WBCs are diploid. Ginkgo was used for DNA copy number analysis. Variability score (VS) quantifies DNA and assay quality (low VS corresponds to high quality). (B) Karyotyping of a matched bone lesion biopsy from patient GU-2, conducted by Caris Life Sciences using whole exome sequencing method and copy number analysis.

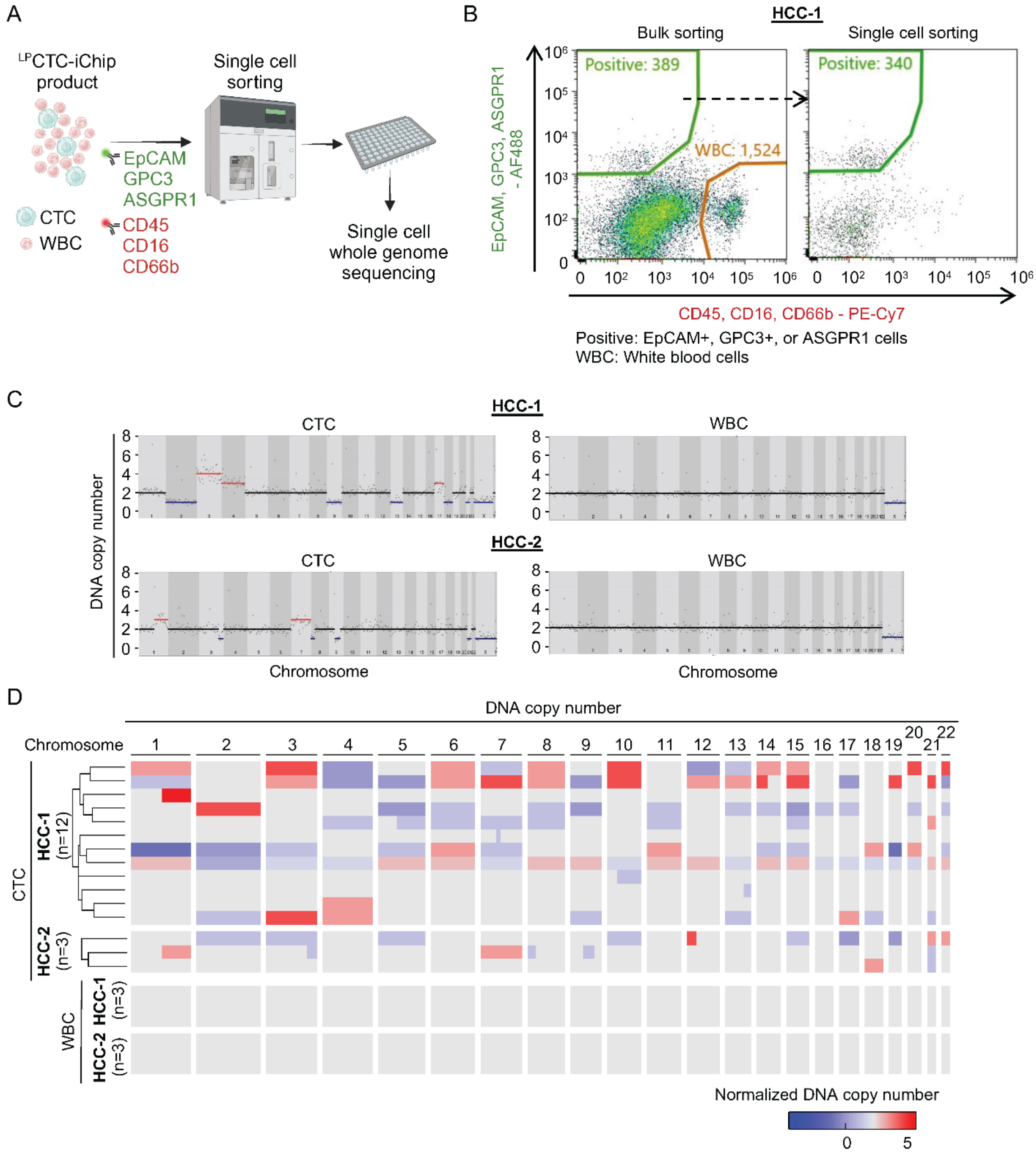

Supplementary Figure 10: Isolation of single CTCs from the diagnostic leukapheresis products of patients with HCC and single-cell DNA copy number analysis. (A) Schematic of single-cell isolation using FACS sorting of LPCTC-iChip-enriched products, derived from leukopak samples of patients with metastatic HCC, followed by single-cell whole genome sequencing. Figure was created using BioRender (Agreement number: RU26ZQODCQ). (B) Two-step-sorting strategy using FACS sorting (SONY sorter): initial bulk sorting into a tube to

remove dead cells and contaminating WBCs is followed by single-cell sorting to isolate individual CTCs into wells on a plate. Sorting is performed using pooled AF488-conjugated antibodies against the epithelial marker EpCAM and the liver-specific markers GPC3 and ASGPR1 versus pooled PE-Cy7-conjugated antibodies against WBC markers CD45, CD16, and CD66b. (C) Representative DNA copy-number variation (CNV) analysis in individual CTCs, compared with diploid WBCs from patients HCC-1 and HCC-2. Ginkgo was used for DNA copy number analysis from single-cell whole genome sequencing data. (D) Unsupervised hierarchical clustering of the normalized heatmap showing individual cell CNVs derived from single-cell whole genome sequencing data of HCC CTCs (n=15) and WBCs. CTCs from HCC-1 show two distinct subpopulations based on CNV analyses. WBCs are shown as negative controls. Ginkgo was used for DNA copy number analysis.

Supplementary Table 1: Patient details and leukapheresis parameters

**Tumor type**	**Tumor stage**	**Total blood volume processed (mL)**	**Duration (minutes)**	**Machine used**	**Blood processing flow rate on the apheresis machine (mL/minute)**
GU-1	Stage IV	6379	109	Spectra Optia	60.0
GU-2	Stage IV	5732	120	Spectra Optia	60.0
TNBC-1	Stage IV	5991	120	Spectra Optia	51.4
HCC-1	Stage IV	4565	107	Spectra Optia	40.0
HCC-2	Stage IVB	7176	120	Spectra Optia	60.0
UM-1	Stage IIIC	6523	120	Spectra Optia	60.0
UM-2	Stage IIIA	4345	120	Spectra Optia	50/32

Supplementary Table 2: List of antibodies for immunofluorescence staining

Antibody	Vendor	Catalog Number	Clone	Working Concentration
EpCAM – AF488	Cell Signaling	5198S	VU1D9	5 µg/ml
Pan-Keratin (C11) – AF488	Cell Signaling	4523S		5 µg/ml
Cytokeratin 19 – AF488	Invitrogen	MA5-18158	A53-B/A2	5 µg/ml
CD16 – AF647	BioLegend	302020	3G8	5 µg/ml
CD45 – AF647	BioLegend	304056	HI30	5 µg/ml
CD66b – AF647	BioLegend	305110	G10F5	5 µg/ml
Sox10 – AF488	Abcam	270150	SP267	5 µg/ml
Melan-A – AF488	Abcam	200544	EP1422Y	5 µg/ml
NG2/MCSP – AF488	R&D Systems	FAB2585G	LHM-2	5 µg/ml
ASGR1 – FITC	Novus Biologicals	NBP1-51109	8D7	5 µg/ml
GPC3 – AF488	Novus Biologicals	NBP2-47763AF488	1G12 + GPC3/863	5 µg/ml
PSMA – AF488	Invitrogen	MA5-18161	GCP-05	5 µg/ml
CD16 – PE-Cy7	BioLegend	980110	3G8	10 µg/ml
CD45 – PE-Cy7	BioLegend	982310	HI30	1.25 µg/ml
CD66b – PE-Cy7	BioLegend	396910	QA17A51	5 µg/ml
LIVE/DEAD™ Fixable Red	Invitrogen	L34971		1:2,000 dilution

Supplementary Data 1: List of ddPCR probes

Supplementary Data 2: Whole exome sequencing analysis revealed variants of clinically unknown significance in CNV-confirmed CTCs from prostate cancer patients.

Supplementary Data 3: Mutations were identified using FDA-approved genetic tests in tissue biopsies and ctDNAs from prostate cancer patients.

Supplementary Data 4: Gene set enrichment analysis for single-cell RNA-seq data of CNV-confirmed CTCs from prostate cancer patients.

Supplementary Data 5: Differential gene expression between EpCAM- and/or PSMA-positive CTCs and DN CTCs from prostate cancer patient GU-2.
For each gene, a two-sample var-equal t-test was run. The resulting p-values were adjusted for multiple hypothesis testing by the Benjamini-Hochberg method (resulting in the "FDR" column) and by the Bonferroni method (resulting in the "FWER" column). Genes for which the fold-change was greater than two and for which the FDR was less than 0.25 were considered differentially expressed.
