# Supplementary material for: Tumor cell-based liquid biopsy using high-throughput microfluidic enrichment of entire leukapheresis product
Source: Nat Commun. 2025 Jan 2;16:32. doi: 10.1038/s41467-024-55140-x (PMC11696112; doi:10.1038/s41467-024-55140-x)
Supplement: Supplementary file 3 — Reporting Summary [file 41467_2024_55140_MOESM3_ESM.pdf]

nature portfolio

Corresponding author(s): Daniel Haber, David Miyamoto, and Mehmet Toner

Last updated by author(s): Aug 31, 2024

Reporting Summary

Nature Portfolio wishes to improve the reproducibility of the work that we publish. This form provides structure for consistency and transparency in reporting. For further information on Nature Portfolio policies, see our Editorial Policies and the Editorial Policy Checklist.

Statistics

For all statistical analyses, confirm that the following items are present in the figure legend, table legend, main text, or Methods section.

n/a	Confirmed	
☐	☒	The exact sample size (*n*) for each experimental group/condition, given as a discrete number and unit of measurement
☐	☒	A statement on whether measurements were taken from distinct samples or whether the same sample was measured repeatedly
☐	☒	The statistical test(s) used AND whether they are one- or two-sided<br>*Only common tests should be described solely by name; describe more complex techniques in the Methods section.*
☒	☐	A description of all covariates tested
☐	☒	A description of any assumptions or corrections, such as tests of normality and adjustment for multiple comparisons
☐	☒	A full description of the statistical parameters including central tendency (e.g. means) or other basic estimates (e.g. regression coefficient) AND variation (e.g. standard deviation) or associated estimates of uncertainty (e.g. confidence intervals)
☐	☒	For null hypothesis testing, the test statistic (e.g. *F*, *t*, *r*) with confidence intervals, effect sizes, degrees of freedom and *P* value noted<br>*Give P values as exact values whenever suitable.*
☒	☐	For Bayesian analysis, information on the choice of priors and Markov chain Monte Carlo settings
☒	☐	For hierarchical and complex designs, identification of the appropriate level for tests and full reporting of outcomes
☒	☐	Estimates of effect sizes (e.g. Cohen's *d*, Pearson's *r*), indicating how they were calculated

Our web collection on statistics for biologists contains articles on many of the points above.

Software and code

Policy information about availability of computer code

Data collection	High-content multispectral immunofluorescence images of cells were acquired at 40X magnification using commercial Vectra Polaris sofiware from the Akoya Biosciences. The images were subsequently exported and processed using a HALO image analysis platform. Cell sorting was conducted using SONY sorter SH800S and data analysis was performed on its cell sorter software for the flow images.
Data analysis	The multispectral immunofluorescence images of cells were analyzed using commercial HALO AI image analysis platform from Indica Labs. Also see the following sections of the Methods section of the supplemental information: WGS pipeline, Copy number heatmap, Variability score, RNA-seq pipeline, Gene Set Enrichment Analysis (GSEA), WES and mutation analysis, ddPCR Data Analysis

For manuscripts utilizing custom algorithms or software that are central to the research but not yet described in published literature, software must be made available to editors and reviewers. We strongly encourage code deposition in a community repository (e.g. GitHub). See the Nature Portfolio guidelines for submitting code & software for further information.

Data

Policy information about availability of data

All manuscripts must include a data availability statement. This statement should provide the following information, where applicable:

- Accession codes, unique identifiers, or web links for publicly available datasets
- A description of any restrictions on data availability
- For clinical datasets or third party data, please ensure that the statement adheres to our policy

RNA-seq raw fastq files and read counts are publicly available at Gene Expression Omnibus (GEO) accession GSE255889. WGS and WES raw fastq files generated in

this study have been deposited in the Open Science Framework (OSF) database. These data are available under restricted access to protect patient information due to IRB policy; access can be obtained upon request to corresponding authors. The processed WGS and WES data are in the Supplementary Data and Source Data files.

Research involving human participants, their data, or biological material

Policy information about studies with human participants or human data. See also policy information about sex, gender (identity/presentation), and sexual orientation and race, ethnicity and racism.

Reporting on sex and gender	Gender of the study participants was collected where provided as self-reporting data and it is outlined in Table 1.
Reporting on race, ethnicity, or other socially relevant groupings	We have not reported data on race, ethnicity, or other socially relevant groupings in this manuscript.
Population characteristics	Detailed description of the cohort characteristics and prior treatment is outlined in Table 1
Recruitment	Patients with breast, liver, prostate cancers and Uveal Melanoma who consented to the MGB IRB protcol # 2020P000251 were included in this study.
Ethics oversight	Leukapheresis on cancer patients was performed at the MGH Apheresis Center using an experimental protocol reviewed and authorized by the MGB Institutional Review Board (MGB IRB 2020P000251). Whole blood donations from healthy donors at our lab were collected using MGB IRB Protocol # 2009-P-000295. In some cases, whole blood samples from healthy donors were also procured from Research Blood Components, LLC (Brighton, MA).

Note that full information on the approval of the study protocol must also be provided in the manuscript.

Field-specific reporting

Please select the one below that is the best fit for your research. If you are not sure, read the appropriate sections before making your selection.

- [x] Life sciences
- [ ] Behavioural & social sciences
- [ ] Ecological, evolutionary & environmental sciences

For a reference copy of the document with all sections, see nature.com/documents/nr-reporting-summary-flat.pdf

Life sciences study design

All studies must disclose on these points even when the disclosure is negative.

Sample size	All available samples were included and no sample size calculation was performed.
Data exclusions	Single cell WGS results were excluded if variability scores were higher than 0.85 and no clear CNV was observed.<br>Single cell RNA-seq results were excluded if the number of aligned reads were less than 300,000 and % of genes aligned to were less than 10%.
Replication	Because data were generated from patients blood samples, the analyses was not replicated.
Randomization	This is an observational study, so no randomization was performed.
Blinding	There was no allocation of subject to groups.

Reporting for specific materials, systems and methods

We require information from authors about some types of materials, experimental systems and methods used in many studies. Here, indicate whether each material, system or method listed is relevant to your study. If you are not sure if a list item applies to your research, read the appropriate section before selecting a response.

Materials & experimental systems

n/a	Involved in the study	
[ ]	[x]	Antibodies
[ ]	[x]	Eukaryotic cell lines
[x]	[ ]	Palaeontology and archaeology
[x]	[ ]	Animals and other organisms
[x]	[ ]	Clinical data
[x]	[ ]	Dual use research of concern
[x]	[ ]	Plants

Methods

n/a	Involved in the study	
[x]	[ ]	ChIP-seq
[ ]	[x]	Flow cytometry
[x]	[ ]	MRI-based neuroimaging

Antibodies

Antibodies used	EpCAM – AF488 Cell Signaling 5198S VU1D9 5 μg/ml<br>Pan-Keratin (C11) – AF488 Cell Signaling 4523S 5 μg/ml<br>Cytokeratin 19 – AF488 Invitrogen MA5-18158 A53-B/A2 5 μg/ml<br>CD16 – AF647 BioLegend 302020 3G8 5 μg/ml<br>CD45 – AF647 BioLegend 304056 HI30 5 μg/ml<br>CD66b – AF647 BioLegend 305110 G10F5 5 μg/ml<br>Sox10 – AF488 Abcam 270150 SP267 5 μg/ml<br>Melan-A – AF488 Abcam 200544 EP1422Y 5 μg/ml<br>NG2/MCSP – AF488 R&D Systems FAB2585G LHM-2 5 μg/ml<br>ASGR1 – FITC Novus Biologicals NBP1-51109 8D7 5 μg/ml<br>GPC3 – AF488 Novus Biologicals NBP2-47763AF488 1G12 + GPC3/863 5 μg/ml<br>PSMA – AF488 Invitrogen MA5-18161 GCP-05 5 μg/ml<br>CD16 – PE-Cy7 BioLegend 980110 3G8 10 μg/ml<br>CD45 – PE-Cy7 BioLegend 982310 HI30 1.25 μg/ml<br>CD66b – PE-Cy7 BioLegend 396910 QA17A51 5 μg/ml<br>LIVE/DEAD™ Fixable Red Invitrogen L34971 1:2,000 dilution
Validation	EpCAM – AF488 antibody was validated internally by testing with CTC cell lines BRx-142, BRx-68 and LNCaP. Healthy donor derived WBCs were used as negative control.<br>Pan-Keratin (C11) – AF488 antibody was validated internally by testing with CTC cell lines BRx-142, BRx-68 and LNCaP. Healthy donor derived WBCs were used as negative control.<br>Cytokeratin 19 – AF488 antibody was validated internally by testing with CTC cell lines BRx-142, BRx-68 and LNCaP. Healthy donor derived WBCs were used as negative control.<br>CD16 – AF647 antibody was validated internally by testing with healthy donor derived WBCs as positive control. Cancer cell lines BRx-142, BRx-68, HepG2, Mel167 and LNCaP were used as negative control.<br>CD45 – AF647 antibody was validated internally by testing with healthy donor derived WBCs as positive control. Cancer cell lines BRx-142, BRx-68, HepG2, Mel167 and LNCaP were used as negative control.<br>CD66b – AF647 antibody was validated internally by testing with healthy donor derived WBCs as positive control. Cancer cell lines BRx-142, BRx-68, HepG2, Mel167 and LNCaP were used as negative control.<br>Sox10 – AF488 antibody was validated internally by testing with cancer cell line Mel167. Healthy donor derived WBCs were used as negative control.<br>Melan-A – AF488 antibody was validated internally by testing with cancer cell line Mel167. Healthy donor derived WBCs were used as negative control.<br>NG2/MCSP antibody was validated internally by testing with cancer cell lines DMS79 and Mel167. Healthy donor derived WBCs were used as negative control.<br>ASGR1 – FITC antibody was validated internally by testing with cancer cell line HepG2. Healthy donor derived WBCs were used as negative control.<br>GPC3 – AF488 antibody was validated internally by testing with cancer cell line HepG2. Healthy donor derived WBCs were used as negative control.<br>PSMA – AF488 antibody was validated internally by testing with cancer cell line LNCaP. Healthy donor derived WBCs were used as negative control.<br>CD16 – PE-Cy7 antibody was validated internally by testing with healthy donor derived WBCs as positive control. Cancer cell lines BRx-142, BRx-68, HepG2, Mel167 and LNCaP were used as negative control.<br>CD45 – PE-Cy7 antibody was validated internally by testing with healthy donor derived WBCs as positive control. Cancer cell lines BRx-142, BRx-68, HepG2, Mel167 and LNCaP were used as negative control.<br>CD66b – PE-Cy7 antibody was validated internally by testing with healthy donor derived WBCs as positive control. Cancer cell lines BRx-142, BRx-68, HepG2, Mel167 and LNCaP were used as negative control.<br>LIVE/DEAD™ Fixable Red was validated internally by testing with 70% ethanol treated cancer cell line LNCaP and BRx68 and healthy donor derived WBCs as positive control. Live cells were used as negative control.

Eukaryotic cell lines

Policy information about cell lines and Sex and Gender in Research

Cell line source(s)	BRx-142, BRx-68, BRx-330, Mel-167 cell lines were internally established at Massachusetts General Hospital by our research group. Cancer cell lines LNCaP, 22Rv1, HepG2 and PC3 were sourced from ATCC.
Authentication	Frequent microscopy checks and growth curve measurements were conducted for cell line authentication. ATCC cell lines were also authenticated periodically by Human Cell STR Profiling Service. Internally established CTC lines have been frequently validated by sequencing for the mutations that were identified in patient tumor biopsies and reported in our previous publications.
Mycoplasma contamination	All cell lines were routinely tested for mycoplasma contamination using MycoAlert kit and results were negative for contamination.
Commonly misidentified lines<br>(See ICLAC register)	NA

Plants

Seed stocks | NA

Novel plant genotypes | NA

Authentication | NA

Flow Cytometry

Plots

Confirm that:

- [x] The axis labels state the marker and fluorochrome used (e.g. CD4-FITC).
- [x] The axis scales are clearly visible. Include numbers along axes only for bottom left plot of group (a 'group' is an analysis of identical markers).
- [x] All plots are contour plots with outliers or pseudocolor plots.
- [x] A numerical value for number of cells or percentage (with statistics) is provided.

Methodology

Sample preparation

The enriched leukopak by LPCTC-iChip were Fc blocked (Jackson ImmunoResearch Laboratories, Cat# 009-000-008) and then immunostained with AF488-conjugated EpCAM (Cell signaling, Cat# 5198) / PSMA (Invitrogen, Cat# MA5-18161) / GPC3 (Novus Biologicals, Cat# NBP2-47763AF488) / FITC-conjugated ASGPR1 (Novus Biologicals, Cat# NBP1-51109) antibodies and PE-Cy7-conjugated CD45 (Biolegend, Cat# 982310) / CD16 (Biolegend, Cat# 980110) / CD66b (Biolegend, Cat# 396910) antibodies, and LIVE/DEAD Red (Invitrogen, Cat# L34971). Cells were treated with DNase I (Worthington Biochemical Corporation, Cat# LS006361) to reduce cell clumping in single-cell suspensions before cell sorting.

Instrument

SONY sorter SH800S

Software

Cell sorter software version 2.1 LE-SH800SZGCPL

Cell population abundance

We sorted 594 individual candidate CTCs from GU-1 based on AF488 staining (EpCAM and/or PSMA) and the absence of PE-Cy7 staining (multiple WBC markers). In addition, we collected 484 “double-negative (DN)” cells, lacking staining for both AF488 and PE-Cy7. For GU-2, we sorted 192 AF488-positive cells and 192 DN cells. These DN cells are abundant in CTC-enriched populations derived by depletion of WBC markers, but their identity is uncertain. From these single-cell collections, we selected 84 single cells from GU-1 (57 AF488-positive, 27 DN) and 173 single cells from GU-2 (115 AF488-positive, 58 DN) for downstream single-cell whole genome sequencing and RNA sequencing. CTCs were determined by CNV and RNA expression.

Gating strategy

After removal of dead cells and cell fragments using Live Dead Red, we performed two-step sorting methods (the ultra-yield bulk sorting followed by the single cell plate sorting with singlet gating) to isolate single viable CTCs (EpCAM or PSMA positive for prostate cancer and EpCAM or GPC3 or ASGPR1 positive for liver cancer) and WBCs (CD45/CD16/CD66b positive) using SONY sorter SH800. The single viable cells were individually sorted into 96-well PCR plates containing the cell lysis buffer for downstream single-cell whole genome sequencing and RNA sequencing.

- [x] Tick this box to confirm that a figure exemplifying the gating strategy is provided in the Supplementary Information.
