# Supplementary material for: Tumor cell-based liquid biopsy using high-throughput microfluidic enrichment of entire leukapheresis product
Source: Nat Commun. 2025 Jan 2;16:32. doi: 10.1038/s41467-024-55140-x (PMC11696112; doi:10.1038/s41467-024-55140-x)
Supplement: Supplementary file 4 — Description of Additional Supplementary Files [file 41467_2024_55140_MOESM4_ESM.pdf]

Description of Additional Supplementary Files

Supplementary Data 1

Description: List of ddPCR probes.

Supplementary Data 2

Description: Whole exome sequencing analysis revealed variants of clinically unknown significance in CNV-confirmed CTCs from prostate cancer patients.

Supplementary Data 3

Description: Mutations were identified using FDA-approved genetic tests in tissue biopsies and ctDNAs from prostate cancer patients.

Supplementary Data 4

Description: Gene set enrichment analysis for single-cell RNA-seq data of CNV-confirmed CTCs from prostate cancer patients.

Supplementary Data 5

Description: Differential gene expression between EpCAM- and/or PSMA-positive CTCs and DN CTCs from prostate cancer patient GU-2.

For each gene a two-sample var-equal t-test was run. The resulting p-values were adjusted for multiple hypothesis testing by the Benjamini-Hochberg method (resulting in the “FDR” column) and by the Bonferroni method (resulting in the “FWER” column). Genes for which the fold-change was greater than two and for which the FDR was less than 0.25 were considered differentially expressed.
